# Impaired consciousness and unilateral limb movement due to acute limb ischemia complicated by acute cerebral infarction: A case report

**DOI:** 10.1097/MD.0000000000035657

**Published:** 2023-10-27

**Authors:** Jun Sato, Tsukasa Yagi, Yusuke Ishii, Rei Hinoura, Ryuta Kajimoto, Tsukasa Kuwana, Nobutaka Chiba, Takeshi Saito, Kosaku Kinoshita

**Affiliations:** a Division of Emergency and Critical Care Medicine, Department of Acute Medicine, Nihon University School of Medicine, Itabashi-ku, Tokyo, Japan; b Department of Cardiology, Nihon University Hospital, Chiyoda-ku, Tokyo, Japan; c Department of Cardiovascular Surgery, Nihon University School of Medicine, Itabashi-ku, Tokyo, Japan; d Department of Neurosurgery, Nihon University School of Medicine, Itabashi-ku, Tokyo, Japan.

**Keywords:** acute limb ischemia, case report, cerebral infarction, motor impairment, thrombectomy

## Abstract

**Rationale:**

The symptoms of impaired consciousness and unilateral motor impairments are a perfect scenario for cerebral infarction, and a physician can easily miss the findings of limb ischemia on the patient paralyzed side even if acute limb ischemia (ALI) occurs on that side. The purpose of this case report is to reiterate the need to suspect ALI in patients with impaired consciousness who cannot complain of symptoms such as abnormal limb paresthesia or pain.

**Patient concerns:**

An 89-year-old woman with impaired consciousness and motor impairment of the left upper and lower extremities was transported to our hospital.

**Diagnoses:**

Brain magnetic resonance imaging showed a suspected cerebral infarction in the posterior circulation; contrast-enhanced computed tomography showed occlusion of the left axillary artery and left femoral artery; and ultrasonography showed occlusion of the right popliteal artery.

**Interventions:**

Cerebral angiography was performed simultaneously with surgical thrombectomy to treat the ALI. Mechanical thrombectomy was not performed for cerebral infarction.

**Outcomes:**

Although motor impairment of the left upper and lower extremities persisted, the patient successfully underwent limb salvage.

**Lessons:**

Both cerebral infarction and ALI require early diagnosis and treatment. This rare case of cerebral infarction complicated by ALI emphasizes the need to avoid missing the signs of ALI in patients with impaired consciousness.

## 1. Introduction

Acute limb ischemia (ALI) is characterized by a sudden decrease in blood flow to the extremities, occurring within 2 weeks of symptom onset, with poor outcomes.^[1]^ The rate of limb amputation in ALI is as high as 15% to 20%,^[1]^ and the mortality rate is 15% to 20%^[2,3]^ due to coexisting complications such as cardiac disease, cerebrovascular disease, and ischemia-reperfusion injury. Most patients experience lower-extremity ischemia, and upper-extremity ischemia is rare (15%–25%).^[4]^ There are a few reports of multiple cerebral infarctions complicated by ALI.^[5]^ Herein, we describe a case of cerebral infarction and ALI in a patient with impaired consciousness and motor impairment of the left upper and lower extremities; early surgical thrombectomy for ALI was performed to salvage the limbs. To our knowledge, there have been no reports of cerebral infarction presenting with impaired consciousness and unilateral upper and lower extremity motor impairment complicated by ALI on the paralyzed side.

## 2. Case presentation

An 89-year-old woman suddenly collapsed and was brought to our hospital with motor impairment of the left upper and lower extremities and impaired consciousness. Physical examination revealed the following: blood pressure, 164/101 mm Hg; heart rate, 101 beats/min; respiratory rate, 25 breaths per min; saturation of peripheral oxygen, 99% with 6 L of oxygen/min via an oxygen mask with a reservoir bag; body temperature, 36.2°C; and Glasgow coma scale score, 6 points (eyes, 1; verbal, 1; motor, 4). She had motor impairment of the left upper and lower extremities (manual muscle test scores of 0 and 3 in the right upper and lower extremities, respectively). Electrocardiography revealed atrial fibrillation. Echocardiography revealed good cardiac contraction and no left ventricular thrombus. The patient medical history included hypertension. The arterial blood lactate level was elevated to 4.3 mmol/L (normal range <1.8 mmol/L) at the time of admission.

**Figure 1.**
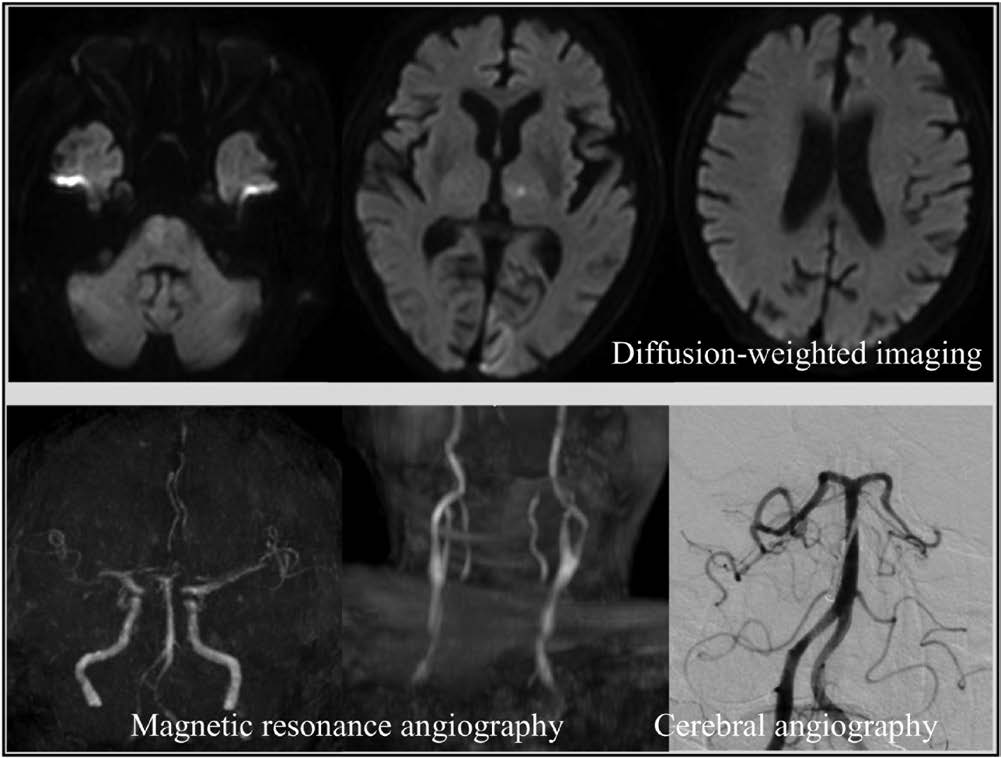
Brain magnetic resonance imaging and cerebral angiography at admission. Brain magnetic resonance imaging reveals scattered acute cerebral infarcts. Brain magnetic resonance angiography shows a suspected occlusion beyond the left P1 segment of the posterior cerebral artery, but cerebral angiography reveals left P2 segment occlusion.

**Figure 2.**
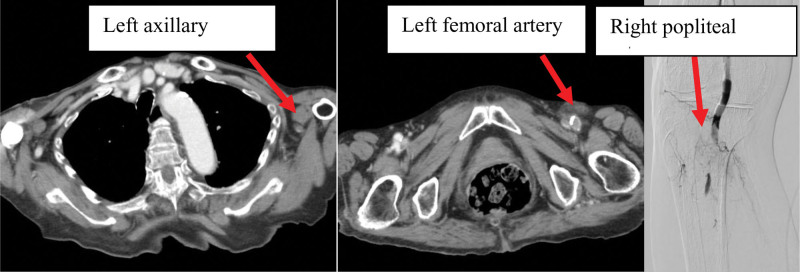
Chest contrast-enhanced computed tomography and lower-extremity angiography at admission. Contrast-enhanced computed tomography and angiography shows obstruction of the left axillary artery, left femoral artery, and right popliteal artery.

Based on these symptoms, we suspected acute cerebral infarction and performed a brain magnetic resonance imaging (MRI). Brain MRI revealed scattered acute cerebral infarcts on diffusion-weighted images in the left cerebellum, pons, left thalamus, left occipital lobe, and both parietal lobes. Brain magnetic resonance angiography revealed a suspected occlusion beyond the left P1 segment of the posterior cerebral artery (PCA) (Fig. 1). Since the left brachial artery and left dorsal pedal artery were pale and not palpable, contrast-enhanced computed tomography was performed, which showed no aortic aneurysm or aortic dissection, and poor contrast in the left axillary and left femoral arteries, leading to the diagnosis of ALI (Fig. 2). Ultrasonography also showed a thrombus in the right popliteal artery, and the right lower extremity was diagnosed with ALI.

Since ALI alone cannot explain the cause of loss of consciousness, cerebral angiography to examine occlusion of the cerebral posterior circulation and surgical thrombectomy for ALI were performed simultaneously. Cerebral angiography showed that the left P2 segment of the PCA was occluded (Fig. 1); however, it was determined to be a peripheral lesion in the posterior circulation and not indicated for mechanical thrombectomy. Simultaneously, surgical thrombectomy using a Fogarty catheter was performed for ALI, which opened the left axillary artery, left femoral artery, and right femoral artery. The time from onset to removal of all thrombi was approximately 6 hours and 25 minutes.

**Figure 3.**
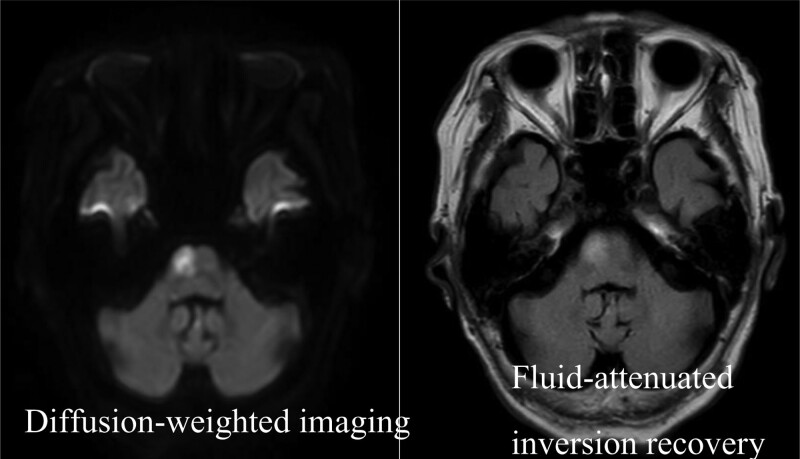
Brain magnetic resonance imaging on hospitalization d 8. Brain magnetic resonance imaging shows cerebral infarction in the pyramidal tract from the pons to the medulla oblongata.

Subsequently, she was put on heparin therapy and switched to oral rivaroxaban on day 6. The left brachial artery and both the dorsal pedal arteries were palpable after surgery; however, the patient motor impairment of the left upper and lower extremities persisted. Her consciousness level improved to a Glasgow coma scale score of 15 (eyes, 4; verbal, 5; motor, 6) on day 2; on day 3, the arterial blood lactate level decreased to 1.7 mmol/L. The peak creatine kinase level during hospitalization was 565 U/L. She did not have compartment syndrome or ischemia-reperfusion injury of the extremities during her hospitalization; however, her motor impairment of the left upper and lower extremities persisted until discharge. On day 8, brain MRI was repeated, which showed high-signal areas on diffusion-weighted imaging and fluid-attenuated inversion recovery in the pyramidal tract from the pons to the medulla oblongata (Fig. 3). On day 12, the patient was transferred to another hospital for rehabilitation.

## 3. Discussion

The symptoms of impaired consciousness and unilateral motor impairments are a perfect scenario for cerebral infarction, and a physician can easily miss the findings of limb ischemia on the paralyzed side. Patients with impaired consciousness cannot complain of abnormal limb paresthesia or pain and should look for signs of ALI. This is a major problem in today medical practice, where we are so focused solely on our specialty that we may ignore other physical examination findings.

The main symptoms of ALI are paresthesia, pain, pallor, pulselessness, poikilothermia, and paralysis.^[1]^ In general, thrombectomy is recommended within 6 hours of the onset of ALI which, otherwise, can lead to irreversible damage to the nerve, muscle, and skin, in that order, within 4 to 6 hours.^[6]^ Nerves and blood vessels often run parallel in the body, and peripheral nerves are mutually nourished by intrinsic and extrinsic blood vessels,^[7]^ so nerves are considered susceptible to ischemia caused by vascular occlusion. Under ischemic conditions due to arterial occlusion, large amounts of reactive oxygen species are produced from endothelial cells in skeletal muscle vessels, causing tissue necrosis. Aerobic processes involved in ATP production within mitochondria are first affected and cellular respiration is completely lost. As a result, energy-hungry cells such as neurons become vulnerable, causing irreversible damage, resulting in severe pain and paresthesia.^[8]^ It has been reported that action potentials of motor axons disappear completely at 46 ± 6 min after the onset of ischemia.^[9]^ In addition, the dysfunction of cell membranes due to ischemia causes albumin to enter the interstitium, forming tissue edema due to its hydrophilic nature. When intramuscular pressure increases owing to this edema, the capillaries collapse and the tissue becomes pale.^[8]^ Thus, we should look for signs of pain, pallor, pulselessness, and poikilothermia in ALI, which do not present in cerebral infarction.

In this case, atrial fibrillation was thought to have caused acute multiple emboli in the left axillary artery, left femoral artery, right popliteal artery, and brain. Therefore, we needed to treat not only ALI but also cerebral infarction at an early stage. In cases of acute cerebral infarction, alteplase administration within 4.5 hours of onset and early mechanical thrombectomy are recommended for acute occlusion of the internal carotid artery or M1 segment of the middle cerebral artery.^[10,11]^ Additionally, mechanical thrombectomy has recently been reported effective for the proximal PCA.^[12]^ In this case, cerebral MRI and magnetic resonance angiography cannot rule out occlusion of the proximal PCA, where mechanical thrombectomy is possible, and cerebral angiography is necessary. There were no contraindications due to the use of alteplase for acute cerebral infarction; however, alteplase was not administered because of the need for surgical thrombectomy for ALI. Therefore, cerebral angiography and surgical thrombectomy for ALI were performed simultaneously by neurosurgeons and vascular surgeons. Cerebral angiography showed no lesions in the posterior circulation that were amenable to thrombus retrieval, and the fact that consciousness improved on day 2 predicted that the cause of the temporary unconsciousness was a temporary occlusion of the basilar artery. The cause of residual motor impairment of the left upper and lower extremities was thought to be cerebral infarction of the right pyramidal tract extending from the pons to the medulla oblongata, as shown on the brain MRI on day 8. Although unilateral upper and lower extremity motor impairment due to cerebral infarction persisted, early diagnosis of ALI and surgical thrombectomy enabled limb salvage.

The outcomes of this study reiterate the importance of finding signs of limb ischemia, such as pulselessness and pallor in the limbs, to look for signs of ALI in patients with impaired consciousness.

A key limitation of this study is the lack of evidence from previous studies recommending simultaneous cerebral angiography and surgical thrombectomy for ALI. Therefore, further studies with more cases are warranted.

## 4. Conclusions

We present a case of early diagnosis of ALI complicated by cerebral infarction and successful surgical thrombectomy in a patient with impaired consciousness and unilateral upper and lower extremity motor impairment, which resulted in limb salvage. This case indicates that the possibility of ALI should be considered when encountering a patient with unilateral extremities motor impairment that cannot be explained by cerebral infarction alone.

## Acknowledgments

We would like to thank the staff of the Intensive Care Unit at Nihon University Hospital.

## Author contributions

**Investigation:** Yusuke Ishii, Rei Hinoura, Ryuta Kajimoto.

**Supervision:** Tsukasa Kuwana, Nobutaka Chiba, Takeshi Saito, Kosaku Kinoshita.

**Writing – original draft:** Jun Sato.

**Writing – review & editing:** Tsukasa Yagi.
